# *In vivo* High Angular Resolution Diffusion-Weighted Imaging of Mouse Brain at 16.4 Tesla

**DOI:** 10.1371/journal.pone.0130133

**Published:** 2015-06-25

**Authors:** Othman I. Alomair, Ian M. Brereton, Maree T. Smith, Graham J. Galloway, Nyoman D. Kurniawan

**Affiliations:** 1 Centre for Advanced Imaging, University of Queensland, Brisbane, Queensland, Australia; 2 School of Pharmacy, University of Queensland, Brisbane, Queensland, Australia; 3 College of Applied Medical Science, King Saud University, Riyadh, Saudi Arabia; National Institute of Radiological Sciences, JAPAN

## Abstract

Magnetic Resonance Imaging (MRI) of the rodent brain at ultra-high magnetic fields (> 9.4 Tesla) offers a higher signal-to-noise ratio that can be exploited to reduce image acquisition time or provide higher spatial resolution. However, significant challenges are presented due to a combination of longer *T*_1_ and shorter *T*_2_/T_2_* relaxation times and increased sensitivity to magnetic susceptibility resulting in severe local-field inhomogeneity artefacts from air pockets and bone/brain interfaces. The Stejskal-Tanner spin echo diffusion-weighted imaging (DWI) sequence is often used in high-field rodent brain MRI due to its immunity to these artefacts. To accurately determine diffusion-tensor or fibre-orientation distribution, high angular resolution diffusion imaging (HARDI) with strong diffusion weighting (b >3000 s/mm^2^) and at least 30 diffusion-encoding directions are required. However, this results in long image acquisition times unsuitable for live animal imaging. In this study, we describe the optimization of HARDI acquisition parameters at 16.4T using a Stejskal-Tanner sequence with echo-planar imaging (EPI) readout. EPI segmentation and partial Fourier encoding acceleration were applied to reduce the echo time (TE), thereby minimizing signal decay and distortion artefacts while maintaining a reasonably short acquisition time. The final HARDI acquisition protocol was achieved with the following parameters: 4 shot EPI, b = 3000 s/mm^2^, 64 diffusion-encoding directions, 125×150 μm^2^ in-plane resolution, 0.6 mm slice thickness, and 2h acquisition time. This protocol was used to image a cohort of adult C57BL/6 male mice, whereby the quality of the acquired data was assessed and diffusion tensor imaging (DTI) derived parameters were measured. High-quality images with high spatial and angular resolution, low distortion and low variability in DTI-derived parameters were obtained, indicating that EPI-DWI is feasible at 16.4T to study animal models of white matter (WM) diseases.

## Introduction

Diffusion-weighted imaging (DWI) [1, 2] allows extensive modelling of microscopic water diffusion to characterise tissue structure. Diffusion tensor imaging (DTI) parameters such as fractional anisotropy (FA), axial diffusivity (AD), radial diffusivity (RD) and mean diffusivity (MD), which is the average of DTI eigenvalues, have become indispensable quantitative tools to study white matter (WM) structures and to determine the efficacy of therapeutic interventions [3]. DTI has been also used to study neurological disease models in the rodent brain [4–7], and spinal cord [8], as well as brain connectivity [9, 10] and development [4, 11].

To study rodent brain microstructure effectively, a high image resolution is required. The typical minimum image resolution for mouse brain is approximately 0.1×0.1×0.1 mm^3^ as compared to 2×2×2 mm^3^ for human brain, i.e. about an 8000 times increase in resolution. High field MRI scanners, operating in the range of 4.7 T to 16.4 T, have become indispensable for small animal imaging. In comparison, standard clinical MRI scanners operate in the range of 0.5 T to 3 T.

There are two major advantages of using ultra-high magnetic field scanners for diffusion-weighted imaging. High-field MRI provides high SNR, allowing faster data acquisition or increased spatial resolution[12]. High-field animal scanners are equipped with strong imaging gradients, essential for high spatial resolution and strong diffusion gradient pulses. However, increased sensitivity to artefacts demand careful optimization of MRI acquisition parameters to deliver highest quality images [13].

Many DWI studies of the mouse brain have been performed using *ex vivo* imaging because they provide high spatial resolution images and are free of motion artefacts compared to live imaging. However, *ex vivo* imaging does not allow longitudinal monitoring of disease progression. In addition, *ex vivo* diffusion is affected by the fixation procedures, and therefore they may show a different specificity compared to *in vivo* DWI [14].

Preclinical rodent *in vivo* DWI data has predominantly been acquired using the spin-echo sequence (SE-DWI) [15–17] due to its greater immunity to magnetic susceptibility at high magnetic field. This sequence, however, is time-consuming and allows a limited number of diffusion encoding directions (6–12 directions) within a reasonable data acquisition time. Therefore, *in vivo* SE-DWI data is best suited to conventional DTI processing and fibre tracking [2]. Other high angular resolution diffusion-weighted imaging (HARDI) techniques require at least 30 diffusion-encoding directions with reasonably high b-values (b>3000s/mm^2^) for accurate measurement of fibre orientation distribution (FOD) and fibre tracking [18, 19]. These requirements for HARDI acquisition can be problematic for *in vivo* DWI, as high b-values result in lower overall SNR, and the increased number of diffusion encoding directions result in a longer acquisition time.

DWI with single-shot echo planar imaging readout (SS-EPI DWI) is widely used in clinical imaging [1, 20]. The use of EPI readout provides several advantages: it reduces the susceptibility to bulk patient motion or physiological movements because the data is acquired in fractions of a second [21, 22]; its short acquisition time allows HARDI acquisition with a large number of diffusion-encoding directions; it can provide a high SNR per unit of scanning time, an advantage for DWI [23, 24].

The application of SS-EPI DWI at ultra-high magnetic fields, especially at 16.4T, has its own challenges. These include a fast signal decay due to shorter *T*_2_ and *T*_2_^*^ relaxation times (the latter due to increased magnetic susceptibility induced inhomogeneity), distortion artefacts (due to poor shimming) and increased chemical shift artefact [25, 26]. DWI with segmented readout EPI (segmented-EPI DWI) divides the k-space into multiple interleaved acquisitions. Segmented-EPI DWI has the advantage of reducing susceptibility to off-resonance artefacts and *T*_2_/*T*_2_* decay times [27] while reducing the demand on pulsed gradient performance compared to SS-EPI DWI [24, 28].

In this work, we describe the optimization of a segmented-EPI DWI sequence to acquire *in vivo* HARDI data of adult C57BL/6 mice at 16.4 T at a high in-plane spatial resolution and within an acceptable acquisition time. Our optimization of the sequence parameters addressed technical challenges of DWI of the rodent brain in an ultra-high magnetic field, including the effect of relaxation times, magnetic susceptibility, motion and chemical shift artefacts.

## Optimization of the HARDI protocol for *in vivo* imaging at high magnetic field

### SE-DWI

The Stjeskal-Tanner spin-echo DWI (SE-DWI) sequence is the preferred imaging technique for *ex vivo* mouse brains because it maximises signal-to-noise ratio and spatial resolution given no specific constraint on experiment time [5, 7, 10]. However, *in vivo* mouse brain imaging is time limited, requiring anaesthesia and consideration of animal wellbeing. SE-DWI is also more susceptible to motion artefacts due to relatively long acquisition times required, resulting in cumulative phase encoding errors [29]. The combination of phase errors and less time for signal averaging result in reduced SNR. To compensate for this limitation, images are often acquired with thicker slices resulting in increased adverse partial volume effects [5, 30]. With such considerations, optimising SE-DWI acquisitions to achieve both high in-plane and slice resolution, as well as a high number of diffusion encoding-directions, is problematic.

An alternative DWI method is the stimulated echo (STE)-DWI sequence. This sequence has been tested at 7 T, but suffers from an inherent 50% reduction in SNR associated with the formation of a stimulated echo [31]. Therefore this sequence was not assessed in this study.

### EPI-Readout

Compared to the spin-echo sequence, EPI is more susceptible to a number of artefacts, such as increased sensitivity to magnetic field inhomogeneity, image blurring, Nyquist ghosting, chemical shift and eddy current artefacts [24, 32].

#### Geometric distortion

The major cause of distortion at high magnetic field is local magnetic field inhomogeneity due to magnetic susceptibility differences between adjacent tissues [32]. EPI, consisting of a sequence of gradient echoes, suffers more severely from susceptibility effects compared to spin-echo sequences, especially with increasing length of echo train and echo spacing due to phase error accumulation [33]. These problems are more pronounced for *in vivo* mouse brain MRI than in human imaging because of the relatively small rodent brain size compared to the affected areas and because of the higher field strengths generally associated with small animal imaging. Pronounced signal loss and distortion is observed around the air cavities of the jaw, ear canals and olfactory bulb, and to a lesser extent, the brain-skull interface [34].

#### Limited spatial resolution

The attainable spatial resolution in a single-shot-EPI is limited as the length of the readout period is constrained by signal decay through *T*_2_ or *T*_2_* processes. As the magnetic field increases, *T*_2_* and *T*_2_ decrease. Therefore, the imaging sequence echo time (TE) must be minimised to allow echo acquisition with sufficient SNR [35].

#### Nyquist ghosting and eddy current artefacts

Nyquist ghosts are caused by EPI gradient readout errors. Any mismatch between the alternating gradients due to *eddy current* effects gives rise to phase errors causing ghosting in the phase-encoding direction [36]. Eddy currents are residual magnetic fields induced by gradient switching. They persist after the gradients are switched off, even in self-shielded gradients, causing image distortion through scaling, shifting and shearing in image slices [37].

The effect of eddy currents in DWI is magnified due to the large diffusion gradients employed. DWI sequences using bipolar diffusion gradients [1, 32] can be used to minimize this problem. However, if high b-values are required, bipolar gradients may require longer echo times [38, 39]. We have noted that image distortion due to eddy current artefacts is negligible in our scanner. Eddy current artefacts can be minimised with careful gradient eddy current compensation (preemphasis) adjustment.

#### Chemical shift artefacts

Chemical shift artefacts are more severe at higher magnetic field with the linear increase in resonance frequency separation of fat and water signals [36]. Nyquist ghosting also deteriorates image quality further in the presence of chemical shift artefacts. Chemical shift artefacts can be minimized using EPI with high receiver bandwidth to reduce the sampling time and consequently the echo spacing, but at the expense of lower SNR. Fat suppression (saturation) techniques are therefore necessary for EPI sequences [40].

### Stejskal-Tanner DWI with segmented EPI readout

A combination of the segmented-EPI DWI sequence [41] with a partial Fourier acquisition and reconstruction [42] is preferred for imaging at high magnetic fields as the TE can be significantly shortened. However, segmented EPI is more sensitive to bulk motion effects compared to single-shot EPI sequences [42, 43]. Higher segmentation factors will result in increased accumulation of phase errors.

Bulk motion results in variations in phase shift between successive echoes resulting in image ghosting and the introduction of diffusion-weighting further complicates the correction of the introduced phase error [44]. Several techniques have been developed to reduce the effect of bulk motion on the quality of the acquired data through multi-shot DWI sequences, such as navigator echoes and cardiac and/or respiratory triggering.

*Navigator echo correction* utilises non-phase encoding echoes before or after the imaging echo to correct phase variations of the acquired imaging echoes. The echo position is used to determine the shifts in k-space so that the data can be re-gridded accordingly [28, 45–47].

*Immobilization* of the subject is usually achieved with general anaesthesia and physical restraint (head mask, tooth bar and tape). Administration of anaesthesia using isoflurane-oxygen mixture inhalation is preferred over intraperitoneal injection because it allows for continuous adjustment according to the condition of the animal throughout the duration of the experiment [48, 49].

*Respiratory monitoring and triggering* during image acquisition reduces propagation of motion artefacts. Sharp inhalation or exhalation or irregular patterns should be avoided. Acquisition should be initiated during a plateau in the breathing cycle of the animal. Respiratory triggering, however, increases the experimental time by factor of approximately two [48, 50, 51].

A summary comparison of technical considerations of the SE-DWI and segmented-EPI-DWI methods is presented in Table 1.

**Table 1:** Comparison of SE-DWI and Segmented-EPI DWI.

SE-DWI	Segmented-EPI DWI
Reduced sensitivity to magnetic susceptibility and geometric distortion artefacts	Prone to distortion artefacts and increased demands on the gradient set
Long acquisition time	Shorter acquisition time allows acquisition of more diffusion-encoding directions or more averaging
Relatively low SNR in diffusion weighted images within a limited experiment time	Suitable image quality to study anatomical structures. Allows thinner slices with more averaging to reduce partial volume effects
Unacceptable experiment due to long experiment times and high SAR at short TR values	Provides reproducible results in a tolerable experiment time

## Materials and Methods

### Animal preparation

All mice were housed and handled in accordance with Queensland Animal Care and Protection Act 2001 and the current NHMRC Australian Code of Practice for the Care and Use of Animals for Scientific Purposes. The use of animals was approved by The University of Queensland's Animal Ethics Committee under the following certificates: CAI/004/11 (Centre for Advanced Imaging) and CIPDD/170/11 (Centre for Integrated Preclinical Drug Development) for the development of diffusion MRI sequence using live mice.

Anaesthesia was induced with 3% isoflurane/oxygen and maintained at 1–1.5%, at a flow rate of 1 L/min during imaging experiments. Small adjustments to the isoflurane concentration were used to maintain the respiratory rate between 60 and 75 beats per minute and the animal body temperature was maintained at 30^°^C using warm water circulation of the MRI gradient cooling system [13, 52].

### Imaging Equipment

MRI data were acquired on a 16.4T vertical wide-bore microimaging system, running Paravision 5.1 (Bruker Biospin, Karlsruhe, Germany), using a micro 2.5 gradient coil and 20 mm SAW volume head coil (M2M Imaging, Brisbane, Australia). To reduce geometrical distortion, the mouse brain was initially shimmed globally using a standard free-induction decay (FID)-based first order and Z^2^ shimming procedure. Then a Bruker Mapshim protocol, which employs magnetic field-map information, was used to optimize the shimming of the whole head volume using the first and second order shims. Finally, a localized shim was performed on a rectangular voxel derived using the Point Resolved Spectroscopy (PRESS) method and placed in the centre and encompassing the entire brain to refine the first and second order shims [52]. The improvement obtained by localized field map shimming is shown in S1 Fig.

### DWI imaging Sequences

#### Segmented-EPI DWI

**Fig 1:**
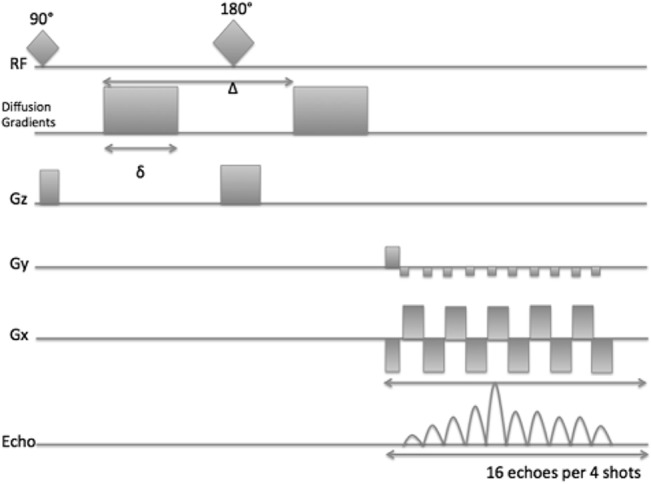
Schematic diagram of segmented-EPI DWI. Segmentation of the echo train is required to reduce off-resonance artefacts. The time between two 90° excitation pulses is the repetition time (TR) and the time from the first 90° excitation pulse to the central echo acquisition is the average echo time (TE). Δ is the separation time between the two applications of diffusion-encoding gradient pulses and δ refers to the duration of the diffusion-encoding gradient. RF (radiofrequency), Gz (slice gradient), Gy (phase gradient), and Gx (readout gradient).

A schematic diagram of the segmented-DWI EPI can be seen in Fig 1. A Bruker Stejskal-Tanner pulse-field gradient spin-echo was interfaced with a segmented EPI read-out sequence to acquire the data with the following parameters: repetition time (TR) = 6000 ms, sampling bandwidth 500 kHz, a minimum echo time (TE) = 13.97 ms to accommodate diffusion gradients with δ/Δ = 2.4/6.4 ms and a b-value of 3000 s/mm^2^. Four dummy scans were employed to ensure steady state conditions. Sixty-four diffusion direction-encoding measurements were acquired within approximately 55 minutes (without respiratory triggering) and 2 hours (with respiratory triggering). The respiratory triggering was required to minimize motion artefacts. Two excitation averages (NEX) were used to increase the SNR, whilst maintaining a reasonable experimental time frame of 2 hours.

**Fig 2:**
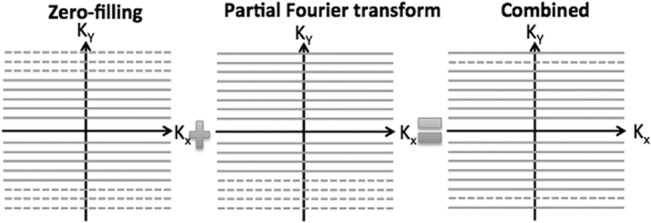
The method of zero-filling and partial Fourier transform. This diagram shows the combination of zero filling and partial Fourier transformation and how they are applied to fill k-space. Zero filling reduces the echo train length and consequently avoids acquisition at late echo period with significant signal decay. Use of this partial Fourier transformation reduces the experiment time by 30%. Dashed lines represent K-space lines, which were not acquired by the combined zero-fill and partial Fourier accelerated acquisition.

MRI data was acquired from 24 contiguous slices acquired at 0.6 mm thickness with FOV = 1.60×0.96 cm and matrix size = 128×64, resulting in an acquired in-plane resolution of 125×150 μm^2^. Partial k-space data was acquired in the phase encoding dimension with a combination of partial Fourier transform (FT) and zero-fill acceleration factors of 1.35 (FT overscans = 15). The encoding acceleration reduced the echo train length (ETL) to avoid acquisition at the late stage of the *T*_*2*_ relaxation period (Fig 2) and the total acquisition time. Encoding acceleration in the frequency-encoding dimension was not used, as it did not reduce TE or the acquisition time. Only moderate partial Fourier and zero filling (truncation of k-space acquisition by approximately 30%) was used to minimize the effect of image smoothing.

The EPI echo train was segmented into 10, 8 and 4 segments to assess the optimal level of segmentation with respect to acquisition time, sensitivity to motion artefacts and reduction of the echo time. Four shot segmentation was found to be optimal. A total of six naïve animals were imaged using the optimized segmented-EPI DWI sequence and the same imaging experiment was repeated twice in four animals.

**Image processing and analysis**: Prior to Fourier transform, the matrix was zero-filled to 256×128, resulting in a final image in-plane resolution of 62.5×75μm^2^. To reduce motion artefacts, diffusion images were registered to a single b_0_ image (image acquired without the application of diffusion gradients) using 2D translation only rigid body registration using the program FSL FLIRT (fsl.fmrib.ox.ac.uk). FA, MD, AD and RD maps were calculated using the MRtrix 0.2.10 program [19].

**Fig 3:**
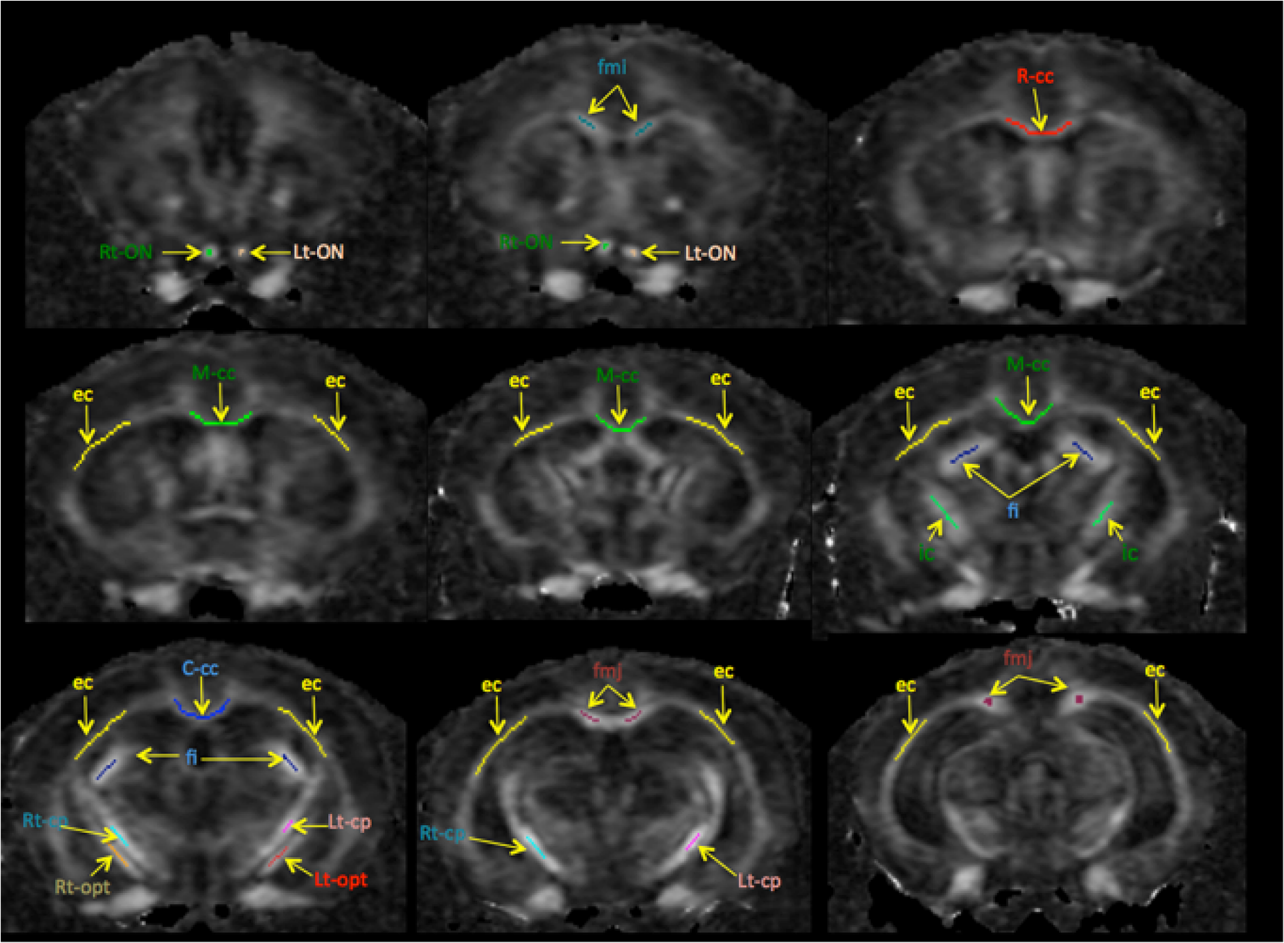
Schematic ROI representations for the measurement of DTI parameters acquired from 2D segmented-EPI DWI *in vivo*. ROI were drawn manually on FA maps of each individual mouse. This image sequence represents rostral (top left) to caudal (bottom right) brain slices. The following structures were analysed: Rt (green) and Lt (brown) optic nerve (ON), forceps minor corpus callosum (fmi) (navy), rostral corpus callosum (R-cc) (red), middle corpus callosum (M-cc) (green), external capsule (ec) (yellow), fimbria (fi) (dark blue), internal capsule (ic) (green), caudal corpus callosum (C-cc) (blue), Rt (navy) and Lt (pink) optic tract (opt), Rt (brown) and Lt (red) cerebral peduncle (cp) and forceps major corpus callosum (fmj) (dark red).

Regions of interests (ROIs) were drawn manually around the white matter (WM) structures on the FA map of each individual mouse using an ITK-snap [53] (Fig 3). The *corpus callosum* was divided into small segments, including forceps minor and major, rostral, middle and caudal. Other WM structures examined included the external capsule, right and left cerebral peduncles, optic tracts, internal capsule and optic nerve, segmented according to the histological atlas [54].

#### Spin-Echo DWI

For comparison with EPI-DWI, spin-echo (SE)-DWI HARDI data (n = 2) were acquired using the following parameters: 24 contiguous slices, 0.6 mm slice thickness, TR/TE = 6000/14.5 ms, b = 3000 s/mm^2^, 30 diffusion-encoding directions and in-plane resolution of 125×150μm^2^, with the acquisition time 2 h 15 mins. Partial Fourier k-space encoding acceleration was applied in both phase- and frequency-encoding dimensions using an acceleration factor of 1.5 (FT overscans = 10) with no zero fill in the frequency direction. These parameters resulted in an acquisition period of 4.5 h if respiratory triggering was employed, which was deemed too long for animal scanning. Thus only sacrificed animals were imaged using these protocols. SE-DW images were processed using the MRtrix program in the same manner as the segmented-EPI DW images [19].

#### Comparison of in vivo segmented-EPI-DWI and in situ SE-DWI

To compare the performance of segmented-EPI DWI and SE-DWI sequences, mice were initially imaged *in vivo* using 2D segmented-EPI DWI sequence. Subsequently, they were sacrificed using an isoflurane overdose inside the scanner and imaged *in situ* using the segmented-EPI DWI sequence followed by the SE-DWI sequence. SNR measurements were obtained from images acquired with and without diffusion gradients. Two ROIs were defined in the central slice package: (1) inside the brain tissue (to measure the signal intensity) and (2) outside the head (to measure the background noise). SNR was calculated as the mean signal intensity of the brain tissue minus the mean signal intensity of the background, divided by the standard deviation of the background [55]. DTI derived parameters (FA, MD, AD and RD) from each condition were also compared.

## Results

### Comparison between *in situ* SE-DWI and *in vivo* segmented EPI-DWI

**Fig 4:**
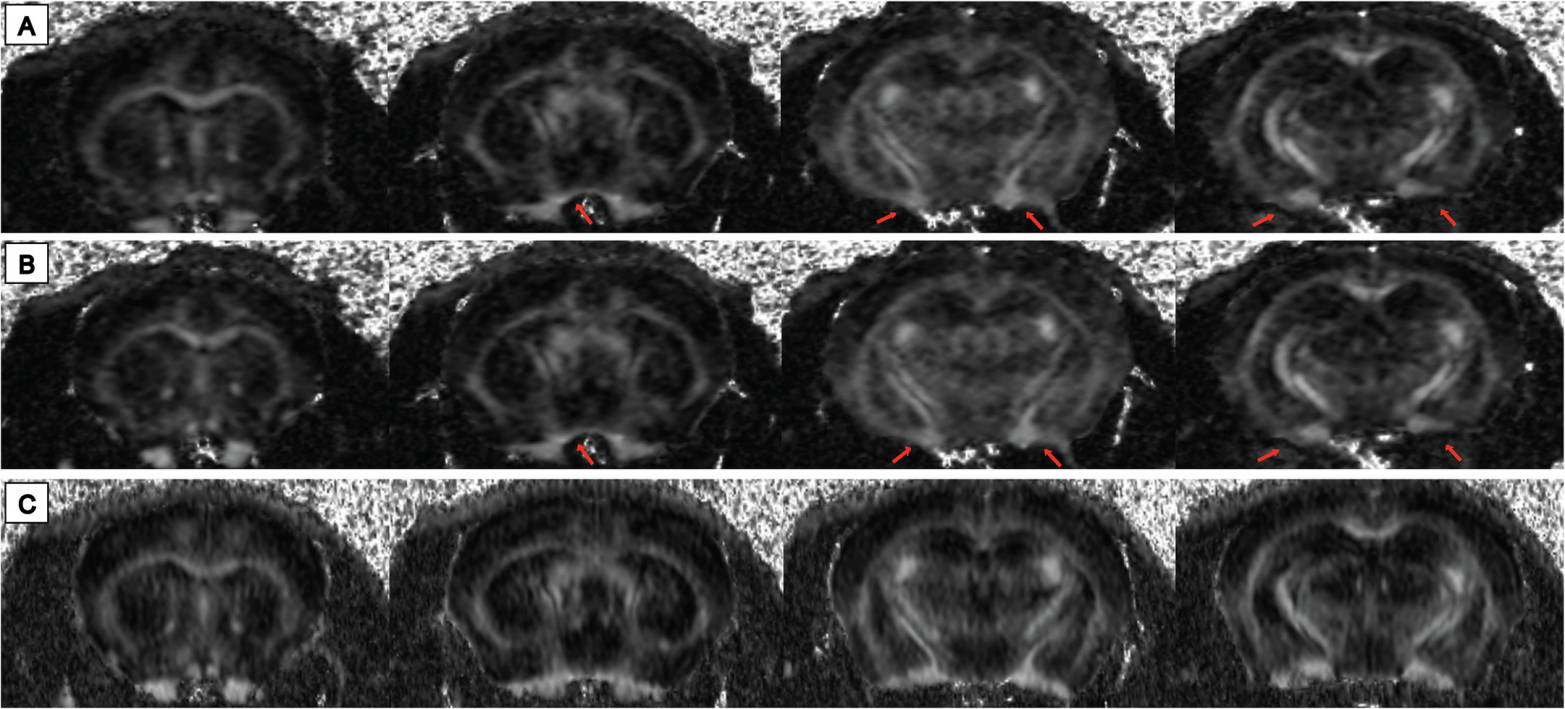
FA map comparison between *in vivo* and in situ segmented EPI-DWI, and *in situ* SE-DWI. Rostral to caudal brain slices of FA maps reconstructed form *in vivo* segmented EPI-DWI (A), *in situ* segmented-EPI DWI (B) and *in situ* SE-DWI (C) acquired at 0.6 mm slice thickness. Distortion artefacts observed in *in vivo* and *in situ* segmented EPI-DWI are shown with red arrows.

SE-DWI was tested to obtain diffusion measurements at 16.4T to enable comparison with the *in vivo* segmented EPI-DWI dataset (Fig 4). Comparisons between SE-DWI and segmented-EPI DWI were made using *in situ* datasets acquired with the same parameters and slice thickness (0.6 mm). *In situ* SE-DWI data showed higher SNR (>36%) compared to *in situ* segmented-EPI DWI. In addition, *in situ* segmented-EPI DWI exhibited 16–20% higher SNR compared to the *in vivo* segmented EPI-DWI data attributable to the absence of motion. Unlike SE DWI, segmented-EPI DWI showed some distortion especially in the ventral brain regions (Fig 4).

DTI derived parameters (FA, MD, AD and RD) of 14 WM brain structures from three DWI acquisitions are available in S1 Table. FA values were smaller in the *in vivo* segmented-EPI DWI compared to the values obtained from *in situ* SE and EPI DWI acquisitions. On the other hand, *in situ* diffusivity parameters are generally smaller than those obtained *in vivo*, presumably due to the absence of blood flow and physiological motion during *in situ* acquisitions [14]. The results of comparison between SE-DWI and segmented-EPI DWI are shown in Table 2.

**Table 2:** Comparison of SE-DWI and Segmented-EPI DWI.

DWI	Segmented-EPI DWI (64 directions)		SE-DWI (30 directions)
	*in vivo* (2h)	*in situ* (1h)	*in situ* (2h15m)
SNR b_0_	29.0 ± 2.3	34.6 ± 1.2	47.4 ± 3.7
SNR b = 3000 s/mm^2^	5.7 ± 1.3	6.8 ± 1.4	12.4 ± 0.9
FA^#^	0.44 ± 0.04	0.50 ± 0.05	0.53 ± 0.04
MD^#^	5.3 ± 0.5	4.0 ± 0.3	4.0 ± 0.3
AD^#^	8.2 ± 0.8	6.4 ± 0.9	6.7 ± 1.1
RD^#^	3.8 ± 0.7	2.8 ± 0.6	2.6 ± 0.5

^#^ Average ± standard deviations of 14 WM structures, the units for MD, AD and RD = 10^−4^ mm^2^/s.

**Fig 5:**
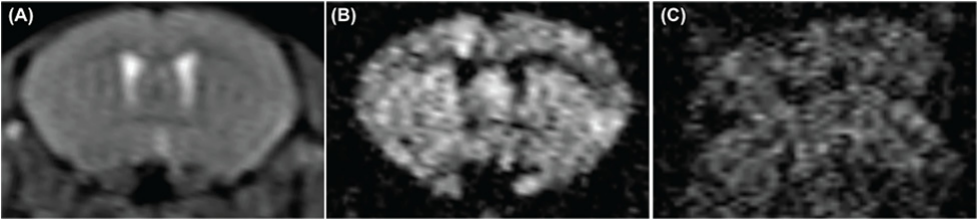
Illustration of signal loss due to motion in *in vivo* segmented EPI DWI. (A) b_0_ image, (B) and (C) DWI images, all are from the same slice position but were acquired in the presence of minimal (A, B) and excessive motion (C).

**Fig 6:**
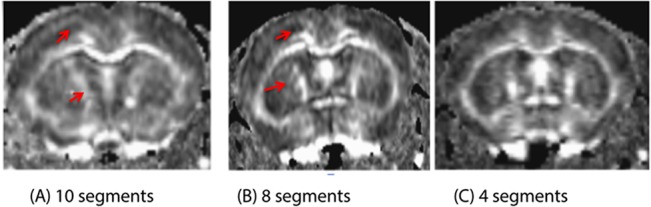
Optimization of ETL segmentation to reduce image artefacts. The FA map from 4-segment EPI-DWI (C) shows less susceptibility to motion artefacts and structure displacement compared the maps reconstructed from 10 and 8-segment ETL (A and B, respectively). This can be observed in the anterior cingulate cortex adjacent to the corpus callosum.

Despite efforts to minimize motion, artefacts may be observed in mouse brain HARDI data (Fig 5). During the implementation of segmented EPI-DWI, a number of ETL segmentation factors were tested (10, 8 and 4) (Fig 6). Increasing number of segments showed more motion artefacts due to more misalignment of k-space lines. Four-segmented ETL produced a good compromise between low image distortion, low sensitivity to motion and reasonable total acquisition time (Fig 6C).

### Diffusion tensor imaging of mouse brain white matter

**Fig 7:**
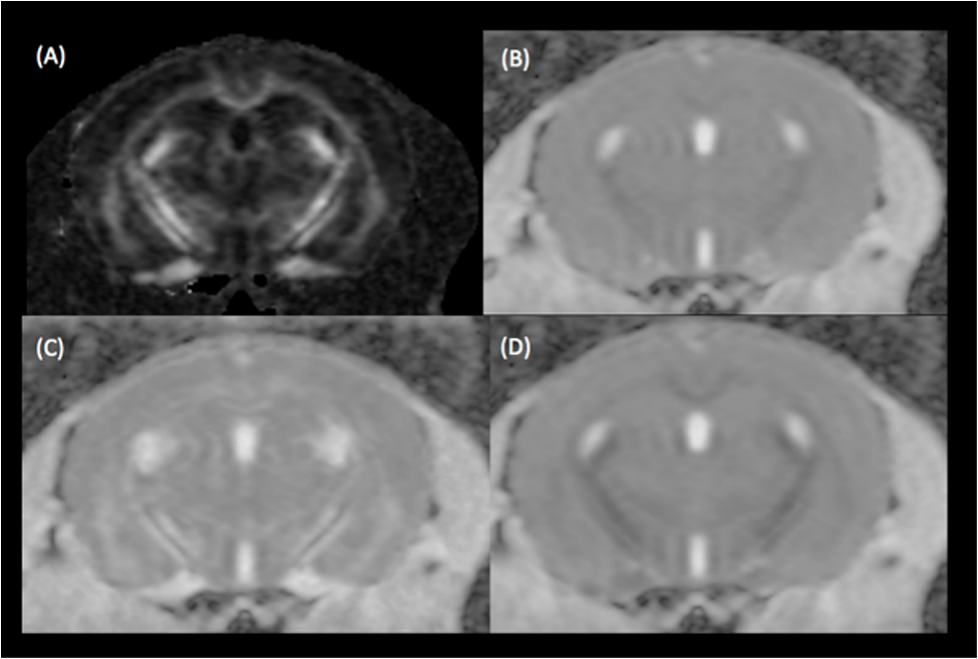
Examples of the DTI derived parameters of the level of the mid-brain structures from *in vivo* 2D EPI DWI at 16.4 T. (A) FA, (B) MD, (C) AD and (D) RD maps.

**Fig 8:**
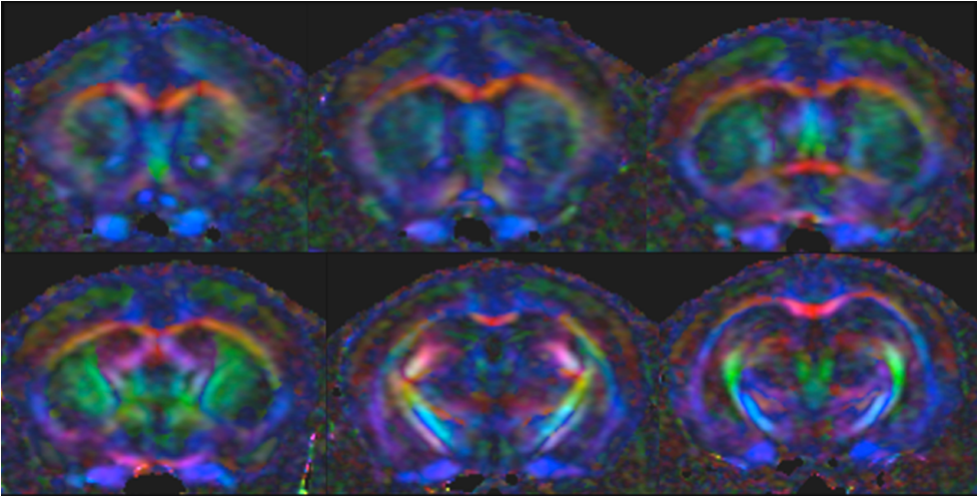
Example of FA colour map of mouse brain from *in vivo* 2D EPI DWI data. Left top to right bottom represent rostral to caudal brain anatomical level, the following directional colour encoding is used: red = medial-lateral, green = rostral-caudal, blue = dorsal-ventral.

**Fig 9:**
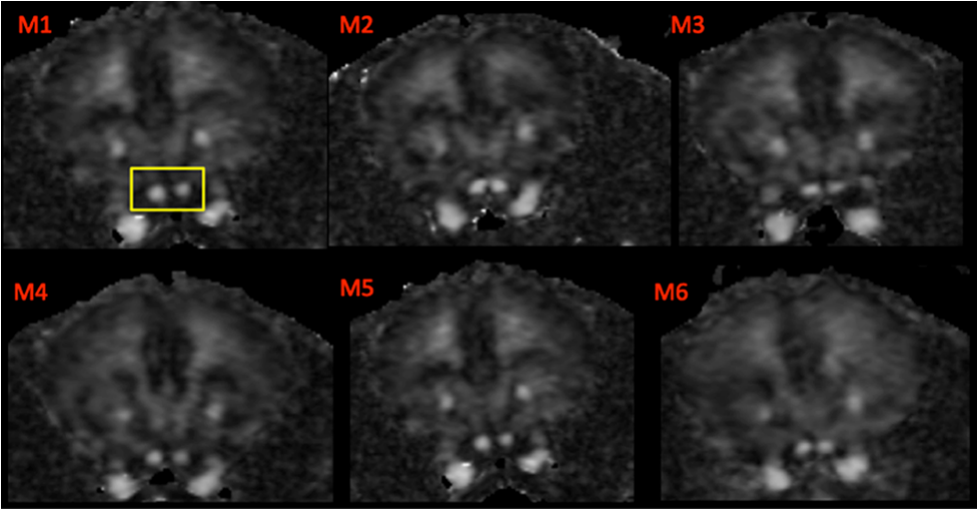
FA maps of the optic nerves from *in vivo* 2D EPI DWI data. Images were reproducible across six mice (M1-M6) and were less susceptible to motion artefacts with reduced partial volume effects in comparison to the SE-DWI experiment. The ROI analysis used only 3–4 voxels in the centre of the nerve to reduce partial volume effects. The optic nerves are the two hyperintense structures inside the yellow box.

Two-dimensional segmented-EPI DWI at 16.4T produced images with good resolution, low distortion and sufficient SNR, suitable for studying white matter structures of the whole brain. Most of the major WM structures can be easily identified, including the corpus callosum, external capsule, cerebral peduncles, optic tracts, optic nerve and fimbria. Representative DTI parametric maps, acquired using *in vivo* 2D segmented-EPI DWI sequence, are shown in Fig 7. Fig 8 shows FA colour maps of the mouse brain from rostral to caudal slices wherein the WM structures were clearly visualized according to their expected fibre directions. The quality of the images was acceptable, even in regions that are problematic, such as the optic nerves (Fig 9).

**Fig 10:**
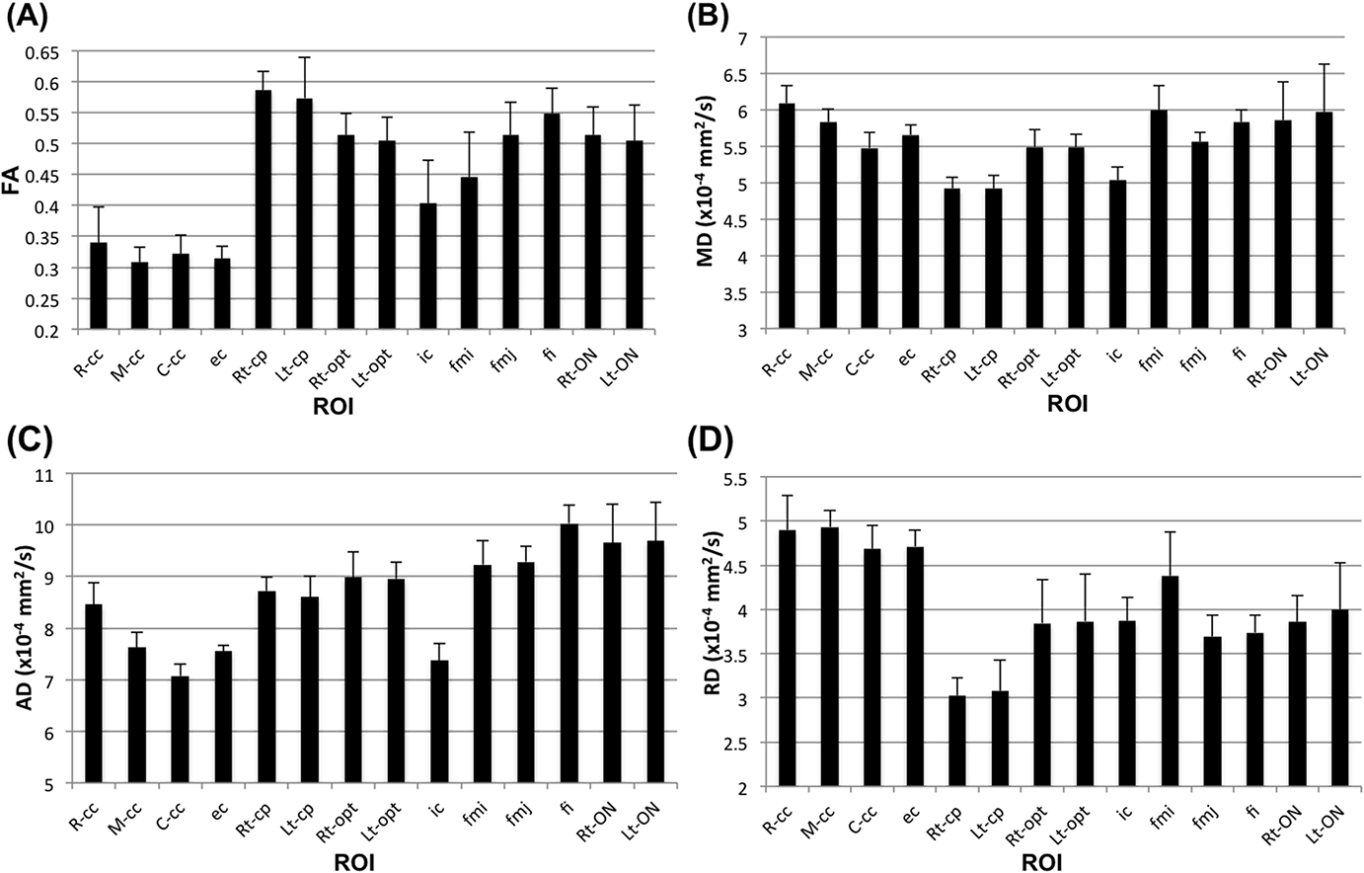
ROI analysis of DTI parameters. (A) FA, (B) MD, (C) AD, and (D) RD calculated from 6 adult wild-type C57BL/6 male mice imaged using *in vivo* segmented 2D DWI-EPI. Data are presented as mean ± standard deviation.

DTI parameters (FA, MD, AD and RD) were analysed in a cohort of 6 wild-type C57BL/6 adult mice (Fig 10). The rostral, middle, caudal and external capsule of the cc were found to have low FA compared to other WM structures, with an average FA of ~0.32. The cerebral peduncle had the highest FA of ~0.57. Other structures such as the optic tracts, the optic nerves, fimbria and the forceps major and minor of the cc had intermediate FA values between 0.4–0.5 (Fig 10A).

The rostral, middle and caudal part of the *corpus callosum* showed an interesting (slowing) pattern of diffusivity, with AD of 8.4, 7.5 and 7.0×10^−4^ mm^2^/s, respectively (Fig 10C). On the other hand, RD was similar for all *cc* segments (4.7–4.9×10^−4^ mm^2^/s; Fig 10D). For RD, the cerebral peduncle has the lowest value at 3×10^−4^ mm^2^/s, whereas other structures such as the optic tract, optic nerve, internal capsule, fimbria, and the forceps of the cc have intermediate RD in the range of 3.7–4.4×10^−4^ mm^2^/s. MD values are variable, ranged between 4.9–6.2×10^−4^ mm^2^/s (Fig 10B).

## Discussion

We have established a suitable protocol for *in vivo* imaging of mouse brain using DWI segmented EPI at 16.4T with excellent image quality for analysing DTI properties of the brain WM structures.

### Considerations for mouse brain *in-vivo* DWI at ultra-high magnetic field

At ultra-high field 16.4T both TR and TE should be optimized to maintain SNR and anatomical contrast. A comparative study of *in vivo T*_1_ relaxation at 9.4T and 17.6T [56] showed only a small increase in *T*_1_ values in the mouse brain. For example, *T*_1_ of the corpus callosum was found to be 1750±50 ms at 9.4T and 1830±90 ms at 17.6T. In implementing *in vivo* DWI of the mouse brain at 16.4T, we measured *T*_1_ and *T*_2_ relaxation times for the cortex (2350±90 and 28±4ms, respectively) and for the corpus callosum (2120±140ms and 23±3ms, respectively), closer to the finding in rat brain *in vivo* at 16.4T [57]. The differences in the measured relaxation may result from differences in the sequence parameters, software acquisition versions and gradient types used in these studies. Nonetheless, long TR (~6s) and short TE (~15ms) values appeared to be important to obtain good EPI DWI datasets at 16.4T.

### Potential application of *in vivo* segmented-EPI DWI

Our experiment demonstrated that the segmented-EPI DWI protocol is sensitive enough to detect distinct normal variations of diffusivities in various regions of the *corpus callosum*. The forceps major and forceps minor regions were found to have higher FA compared to the rostral, middle and caudal cc segments. Additionally, a gradual decline in AD and MD values were detected in these regions. This observation supports the finding that in corpus callosum axon diameters are approximately 20% larger in the rostral-cc compared to the caudal-cc [58]. Such sensitivity is important for studying rodent models of neurological diseases especially those involving the cc.

Our *in vivo* segmented-EPI DWI protocol also produced surprisingly good quality images of the optic nerve. This structure is difficult to image because the diameter of the optic nerve in rodents is only 0.3–0.4 mm. As a result it can be severely affected by imaging artefacts such as motion and local magnetic inhomogeneity due to the proximity to the skull and nose air pockets [59]. *In vivo* assessment of the optic nerve was shown to be valuable for exploring the pathological changes of several neurological diseases [59] such as retinal ischemia [60] and multiple sclerosis [15].

Our DWI protocol aimed to obtain the highest possible spatial and angular resolution and high b-value while maintaining reasonable SNR within an acceptable acquisition time. These constraints unfortunately result in the use of a relatively large 2D slice thickness with accompanying partial volume contributions in some anatomical structures. The effect of partial volume errors on statistical analyses may be reduced using ROIs placed in the centre of WM structures. Low anisotropy structures, such as cortical GM structures and thalamus, have low DTI contrast, and thus accurate segmentation of these structures is difficult. Therefore, reliable assessments using this protocol may be limited to detect pathological changes in major WM structures with high anisotropy to maintain a high level of confidence in the measurements.

### Comparison with other *in vivo* DWI acquisitions

Segmented EPI with a partial Fourier transformation has been previously used at 9.4 T to acquire a non-isotropic 2D DWI dataset (30 diffusion gradient directions, b value = 1000 s/mm^2^, spatial resolution of 156×156 μm^2^, by 500 μm slice thickness) [52]. Our protocol improves the angular resolution to 64 directions, higher b value (3000 s/mm^2^) and in-plane spatial resolution to 125×150 μm^2^, although used thicker slices at 600 μm. The higher SNR at 16.4T allowed a greater diffusion angular resolution and higher diffusion weighting; both are generally desirable for improving the accuracy of the DTI-derived parameters and the ability to resolve crossing-fibres [19, 30, 61]. However, using EPI DWI, the overall resolution achieved in the two studies are similar, as higher matrix size results in long TE, which needs to be avoided due to the short *T*_2_* at 16.4T.

Despite using a large number of diffusion directions, fibertracking using this 2D EPI DWI protocol produced unsatisfactory results (not shown). The streamlines appeared jagged and discontinuous, especially when compared to *ex-vivo* HARDI using the same scanner (3D SE-DWI, 100 μm isotropic resolution, 30 directions, b-value 5000 s/mm^2^, 16h acquisition) [7]. Low streamline quality of the *in vivo* data is most likely originating from: (1) lower resolution and highly anisotropic voxel size resulting in tracing problems in curved WM tracts, and (2) generally lower SNR compared to the *ex-vivo* data.

Our data showed that FA, AD and RD of the cerebral peduncles, optic tracts and fimbria are similar to the previously published study using 3D DWI gradient and spin echo (GRASE) acquisition [6]. It was reported that the FA values of these respective structures were 0.54±0.1, 0.57±0.1 and 0.62±0.09 respectively (data at 9.4T, 3D resolution 117×125×375μm^3^, b-value 1000 s/mm^2^, 6 diffusion-encoding direction). In comparison, our FA data for these respective structures were 0.59±0.07, 0.51±0.04 and 0.55±0.04. Despite differences in acquisition parameters and image resolution, the FA values obtained are similar. Sensitivity to imaging parameters may be reduced in this case due to the predominant direction of the WM fibres in these structures. These fibres run in a rostro-caudal direction, similar to the direction of the stacking of the 2D slices, and thus could minimize the partial volume effect from the thick slices.

For regions within the corpus callosum and external capsule, the FA values determined in our studies were lower compared to a previous study [6]. Besides partial volumes and fibre orientation effects, these differences may be related to different segmentation of corpus callosum ROIs. In our experiment, the corpus callosum was segmented into four regions: rostral, middle, caudal, forceps minor and forceps major producing FA values of 0.34±0.06, 0.31±0.03, 0.32±0.03, 0.45±0.07 and 0.51±0.05, respectively. In the previous study, the corpus callosum was divided into three segments; rostral, middle and caudal with FA values of 0.62±0.11, 0.51±0.09 and 0.59±0.10, respectively [6].

### Improvements of *in vivo* mouse brain DWI acquisition

#### Cryogenic Probe

The availability of cryocoils [62, 63] has significant potential for improving DWI through SNR enhancement by a factor of three [55]. The study by Muller *et al*. at 11.7T showed the feasibility of acquiring DW images (30 diffusion gradients, 156×156×250μm, b-value 1000s/mm^2^) without respiratory triggering in 30 minutes. The cryoprobe allows imaging using much thinner slices, which would be critical for accurate fibertracking and increasing the accuracy of DTI derived parameters [55].

#### Combination of GRASE and cryogenic probe

A combination of 3D DWI GRASE at 11.7T and the cryocoil using moderate angular resolution (12 gradient directions) produced images with high spatial 3D isotropic resolution (125 μm) in 2–2.5 hours [9]. When spatially selective radiofrequency pulses were also used, a higher angular resolution (30 diffusion directions) can be achieved within 1 hour [64], allowing focused imaging of specific brain structures such as hippocampus, motor and sensory cortex [64]. However, whole brain DWI using the same imaging parameters would require approximately 10h [9]. Nevertheless, this development could be extremely beneficial for detailed studies involving diseases affecting specific areas such as focal traumatic brain injury [65] and stroke [66].

## Conclusion

In summary, we have addressed technical challenges to establish a protocol suitable for segmented EPI DWI of whole mouse brain at 16.4 T. The acquired data produced high quality spatial and angular resolution images in a reasonable experiment time (2h with respiratory triggering). ROI analysis shows low variability in the measured DTI-derived parameters in a wildtype animal cohort, indicating that this protocol will be suitable for longitudinal study of animal models of neurological disease, in particular those suffering from WM changes.

## Supporting Information

S1 Fig Field improvement obtained using Mapshim. (A) Magnitude image, (B) field map obtained after FID shimming (step 1), (C) field map obtained after the final localized Mapshim protocol using a large PRESS voxel placed in the centre of the brain (step 3). Brain field histograms were measured using outlined brain areas (ROI_1) before (D) and after (E) the localized Mapshim procedure. The brain local field homogeneity in (D) and (E) was improved from -192±151Hz to 4.15±142 Hz (mean±stdev). Panel group F1-4 and G1-4 are two slices obtained before (F1-2, G1-2) and after (F3-4, G3-4) the localized Mapshim procedure. F1, F3, G1 and G3 are b_0_ images, F2, F4, G2 and G4 are DWI with the same diffusion direction obtained using the optimized DWI segmented EPI sequence. Examples for the improvement of the quality of brain structure definition in the DW images are shown using white arrowheads.
(TIF)

S1 Table DTI derived parameters of *in vivo* and *in situ* segmented-EPI DWI and *in situ* SE-DWI. (DOCX)
