# Supplementary figures and images for: In vivo High Angular Resolution Diffusion-Weighted Imaging of Mouse Brain at 16.4 Tesla
Source: PLoS One. 2015 Jun 25;10(6):e0130133. doi: 10.1371/journal.pone.0130133 (PMC4482319; doi:10.1371/journal.pone.0130133)

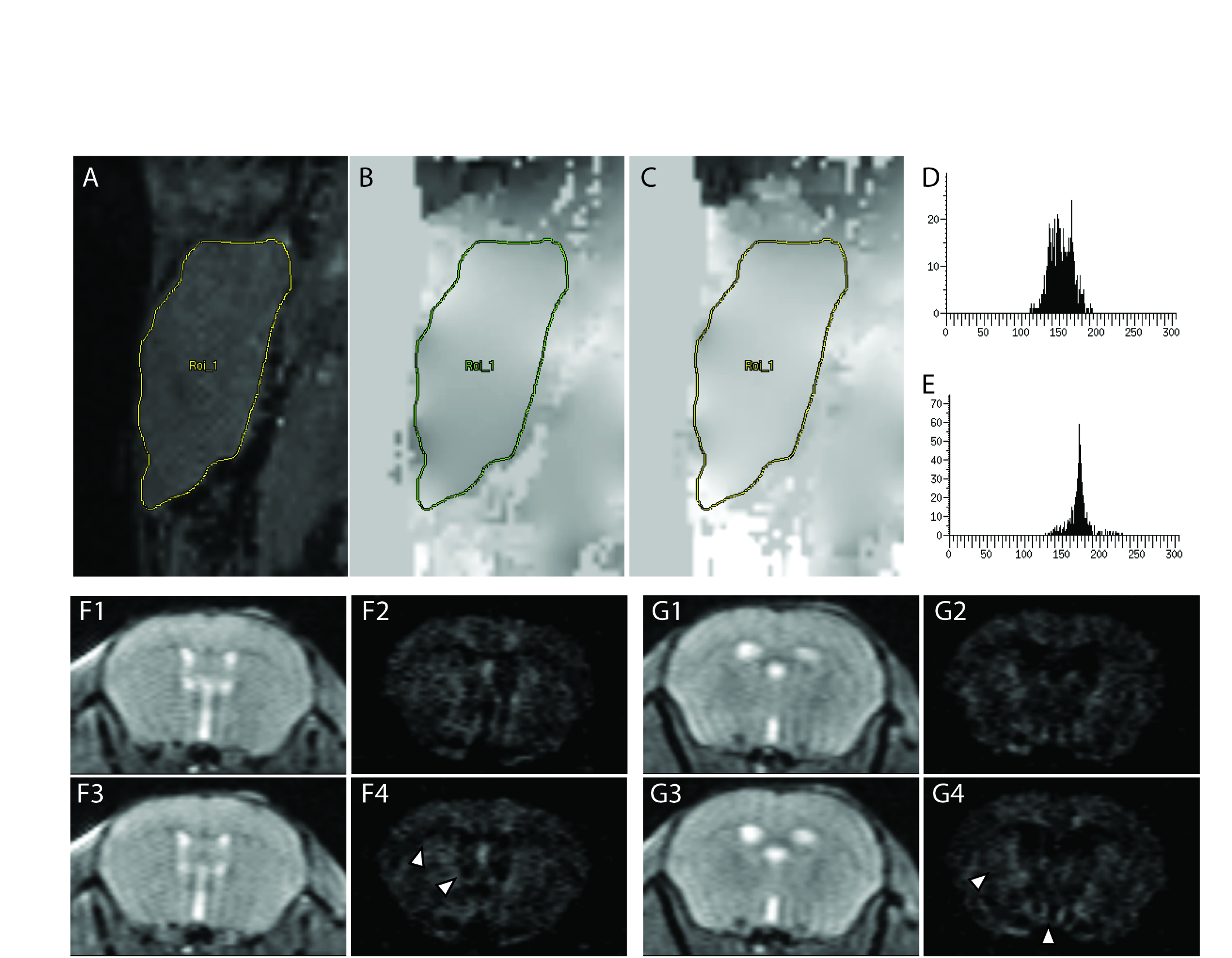

Supplement: S1 Fig — (A) Magnitude image, (B) field map obtained after FID shimming (step 1), (C) field map obtained after the final localized Mapshim protocol using a large PRESS voxel placed in the centre of the brain (step 3). Brain field histograms were measured using outlined brain areas (ROI_1) before (D) and after (E) the localized Mapshim procedure. The brain local field homogeneity in (D) and (E) was improved from -192±151Hz to 4.15±142 Hz (mean±stdev). Panel group F1-4 and G1-4 are two slices obtained before (F1-2, G1-2) and after (F3-4, G3-4) the localized Mapshim procedure. F1, F3, G1 and G3 are b0 images, F2, F4, G2 and G4 are DWI with the same diffusion direction obtained using the optimized DWI segmented EPI sequence. Examples for the improvement of the quality of brain structure definition in the DW images are shown using white arrowheads. (TIF) [file pone.0130133.s001.tif]
