# Supplementary material for: In vivo High Angular Resolution Diffusion-Weighted Imaging of Mouse Brain at 16.4 Tesla
Source: PLoS One. 2015 Jun 25;10(6):e0130133. doi: 10.1371/journal.pone.0130133 (PMC4482319; doi:10.1371/journal.pone.0130133)
Supplement: S1 Table — (DOCX) [file pone.0130133.s002.docx]

**S2 Table. DTI derived parameters of *in vivo* and *in situ* DWI-segmented EPI and *in situ* SE DWI**

| ROI | *In vivo* EPI DWI | | | | *In situ* EPI DWI | | | | *In situ* SE DWI | | | |
| --- | --- | --- | --- | --- | --- | --- | --- | --- | --- | --- | --- | --- |
|  | FA | MD^#^ | AD^#^ | RD^#^ | FA | MD^#^ | AD^#^ | RD^#^ | FA | MD^#^ | AD^#^ | RD^#^ |
| R-cc | 0.30±0.02 | 6×10^-04^± 6 ×10^-05^ | 7.9×10^-04^± 8×10^-05^ | 5×10^-04^± 4.9×10^-05^ | 0.31±0.04 | 4.2×10^-04^± 3.5×10^-05^ | 5.6×10^-04^± 4.8×10^-05^ | 3.6×10^-04^± 3.4×10^-05^ | 0.34±0.04 | 4.2×10^-04^  ±2.7×10^-05^ | 5.7×10^-04^± 5.4×10^-05^ | 3.4×10^-04^± 2.4×10^-05^ |
| M-cc | 0.31±0.03 | 5.6×10^-04^± 2 ×10^-05^ | 7.2×10^-04^± 3.1×10^-05^ | 4.8×10^-04^± 2.5×10^-05^ | 0.35±0.05 | 4.1×10^-04^±  2.1×10^-05^ | 5.5×10^-04^± 5.2×10^-05^ | 3.5×10^-04^± 2.7×10^-05^ | 0.35±0.04 | 4.1×10^-04^  ±3.3×10^-05^ | 5.5×10^-04^± 4.4×10^-05^ | 3.4×10^-04^± 3.4×10^-05^ |
| C-cc | 0.32±0.04 | 4.9×10^-04^± 2 ×10^-05^ | 7×10^-04^± 3.4×10^-05^ | 3.8×10^-04^± 2.7×10^-05^ | 0.35±0.03 | 4.1×10^-04^±  1.8×10^-05^ | 5.4×10^-04^± 3.3×10^-05^ | 3.4×10^-04^± 2.6×10^-05^ | 0.35±0.03 | 4.1×10^-04^± 1.4×10^-05^ | 5.5×10^-04^± 3.5×10^-05^ | 3.4×10^-04^± 2×10^-05^ |
| ec | 0.28±0.05 | 5.5×10^-04^± 3 ×10^-05^ | 7.1×10^-04^± 6.1×10^-05^ | 4.7×10^-04^± 2.8×10^-05^ | 0.35±0.05 | 4.3×10^-04^±  3.0×10^-05^ | 5.9×10^-04^± 5×10^-05^ | 3.5×10^-04^± 3.6×10^-05^ | 0.33±0.07 | 4.2×10^-04^± 2.7×10^-05^ | 5.7×10^-04^± 5.6×10^-05^ | 3.5×10^-04^± 3.8×10^-05^ |
| Rt-cp | 0.64±0.05 | 4.5×10^-04^± 2 ×10^-05^ | 8.4×10^-04^± 5.6×10^-05^ | 2.6×10^-04^± 2.2×10^-05^ | 0.63±0.01 | 3.6×10^-04^±  3.8×10^-05^ | 6.6×10^-04^± 3.1×10^-05^ | 2.1×10^-04^± 5.6×10^-05^ | 0.59±0.03 | 3.5×10^-04^± 2.5×10^-05^ | 6.2×10^-04^± 4.1×10^-05^ | 2.2×10^-04^± 2.1×10^-05^ |
| Lt-cp | 0.51±0.06 | 4.5×10^-04^± 4 ×10^-05^ | 8×10^-04^± 7.5×10^-05^ | 2.7×10^-04^± 3.2×10^-05^ | 0.53±0.03 | 3.6×10^-04^±  5.0×10^-05^ | 6.2×10^-04^± 5.6×10^-05^ | 2.3×10^-04^± 6.6×10^-05^ | 0.58±0.04 | 3.7×10^-04^  ±2.2×10^-05^ | 6.4×10^-04^± 4.8×10^-05^ | 2.3×10^-04^± 1.6×10^-05^ |
| Rt-opt | 0.49±0.02 | 5×10^-04^± 3 ×10^-05^ | 8×10^-04^± 4.7×10^-05^ | 3.5×10^-04^± 9.7×10^-06^ | 0.54±0.03 | 4.6×10^-04^±  2.1×10^-05^ | 7.7×10^-04^± 2.7×10^-05^ | 3.0×10^-04^± 2.3×10^-05^ | 0.66±0.02 | 4.7×10^-04^ ±1.8×10^-05^ | 8.9×10^-04^± 4.9×10^-05^ | 2.6×10^-04^± 1.1×10^-05^ |
| Lt-opt | 0.48±0.05 | 5×10^-04^± 2 ×10^-05^ | 7.9×10^-04^± 2.5×10^-05^ | 3.5×10^-04^± 4.1×10^-05^ | 0.57±0.04 | 4.2×10^-04^±  2.5×10^-05^ | 7.2×10^-04^± 5×10^-05^ | 2.7×10^-04^± 2.4×10^-05^ | 0.60±0.06 | 4.2×10^-04^ ±1.8×10^-05^ | 7.4×10^-04^± 4.1×10^-05^ | 2.6×10^-04^± 3×10^-05^ |
| ic | 0.31±0.03 | 4.9×10^-04^± 3×10^-05^ | 7.6×10^-04^± 3.3×10^-05^ | 3.6×10^-04^± 3.1×10^-05^ | 0.37±0.03 | 3.7×10^-04^±  1.7×10^-05^ | 5.3×10^-04^± 2.5×10^-05^ | 2.9×10^-04^± 2×10^-05^ | 0.48±0.04 | 3.5×10^-04^ ±1.8×10^-05^ | 5.6×10^-04^± 3×10^-05^ | 2.5×10^-04^± 1.9×10^-05^ |
| fmi-cc | 0.38±0.02 | 5.7×10^-04^± 8 ×10^-05^ | 8.3×10^-04^± 1.3×10^-04^ | 4.4×10^-04^± 5.5×10^-05^ | 0.49±0.06 | 4.0×10^-04^±  2.5×10^-05^ | 6.4×10^-04^± 3.9×10^-05^ | 2.8×10^-04^± 3.1×10^-05^ | 0.53±0.03 | 3.6×10^-04^ ±2.1×10^-05^ | 6.1×10^-04^± 4.6×10^-05^ | 2.4×10^-04^± 1.3×10^-05^ |
| fmj-cc | 0.51±0.03 | 5.4×10^-04^± 3 ×10^-05^ | 8.9×10^-04^± 5.4×10^-05^ | 3.7×10^-04^± 2.3×10^-05^ | 0.62±0.05 | 4.0×10^-04^±  2.1×10^-05^ | 7.4×10^-04^± 5.9×10^-05^ | 2.4×10^-04^± 2×10^-05^ | 0.62±0.06 | 4.2×10^-04^ ±2.9×10^-05^ | 7.8×10^-04^± 9×10^-05^ | 2.4×10^-04^± 1.8×10^-05^ |
| fi | 0.55±0.06 | 5.5×10^-04^± 4×10^-05^ | 9.4×10^-04^± 4.3×10^-05^ | 3.5×10^-04^± 5×10^-05^ | 0.70±0.05 | 4.0×10^-04^±  3.2×10^-05^ | 7.9×10^-04^± 5.7×10^-05^ | 2.1×10^-04^± 3.2×10^-05^ | 0.70±0.04 | 3.9×10^-04^± 1.8×10^-05^ | 7.8×10^-04^± 3.9×10^-05^ | 2.0×10^-04^± 2×10^-05^ |
| Rt-ON | 0.52±0.01 | 5.6×10^-04^± 2 ×10^-05^ | 9.2×10^-04^± 3.3×10^-05^ | 3.7×10^-04^± 1.3×10^-05^ | 0.60±0.02 | 3.7×10^-04^±  4.5×10^-06^ | 6.7×10^-04^± 1.1×10^-05^ | 2.2×10^-04^± 1.1×10^-05^ | 0.69±0.04 | 4.1×10^-04^± 3.6×10^-05^ | 8.1×10^-04^± 4.9×10^-05^ | 2.2×10^-04^± 3.3×10^-05^ |
| Lt-ON | 0.51±0.03 | 6.0×10^-04^± 3 ×10^-05^ | 9.8×10^-04^± 5.3×10^-05^ | 4×10^-04^± 2.8×10^-05^ | 0.50±0.05 | 3.7×10^-04^±  2.3×10^-05^ | 6.5×10^-04^± 3.5×10^-05^ | 2.2×10^-04^± 2×10^-05^ | 0.63±0.04 | 4.0×10^-04^ ±4.2×10^-05^ | 7.6×10^-04^± 7.9×10^-05^ | 2.2×10^-04^± 2.8×10^-05^ |

^#^ The units for MD, AD and RD = mm^2^/s
